# Supplementary material for: Three-Year Longitudinal Analysis of Splicing Changes in Myotonic Dystrophy Type 1
Source: bioRxiv. 2026 Oct 1:2026.09.25.754454. Preprint. [Version 1] doi: 10.64898/2026.09.25.754454 (PMC13648444; doi:10.64898/2026.09.25.754454)
Supplement: Supplementary file 1 [file media-1.pdf]

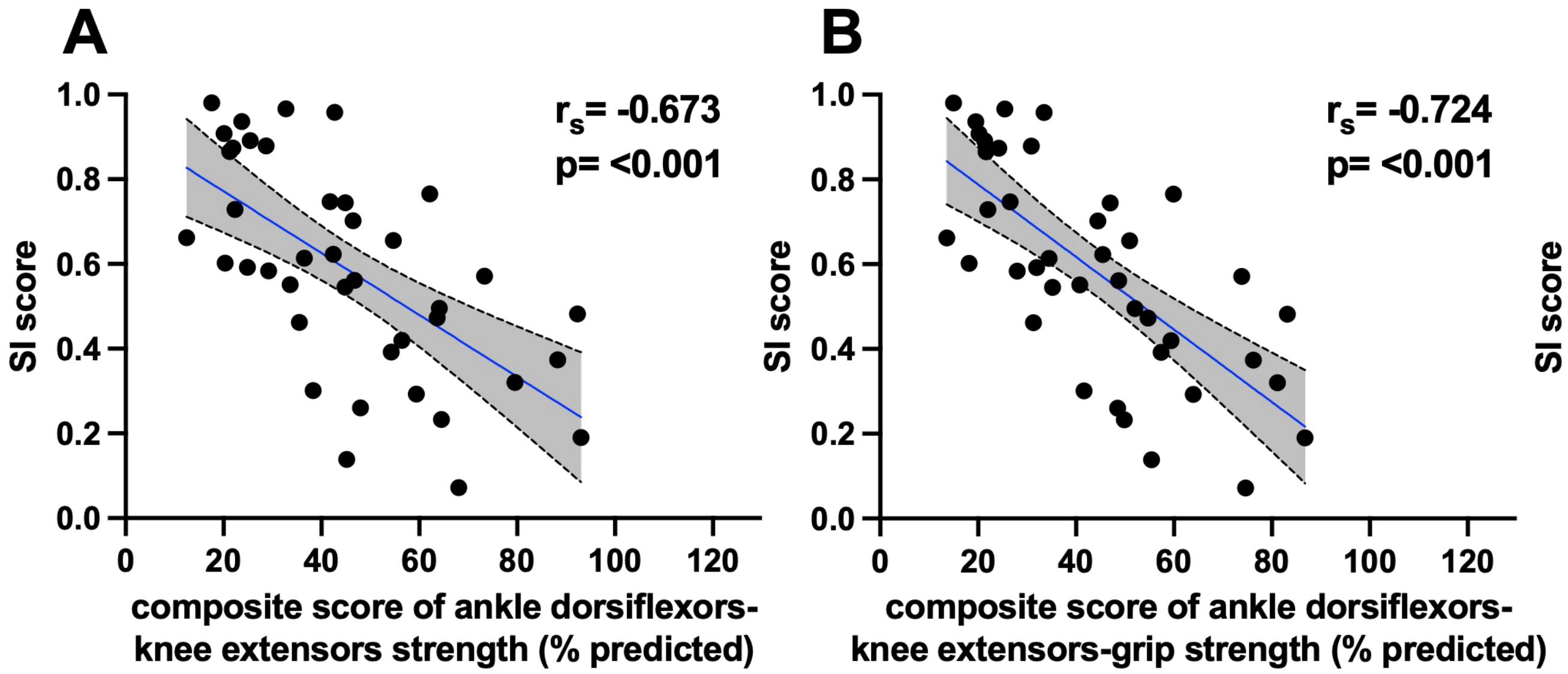


**Supplemental Figure 1: Spearman correlations of splicing index and composite scores in Provenzano et al dataset to validate the approach. A.** Correlation of splicing index and composite score average of percent predicted quantitative muscle testing of ankle dorsiflexors and knee extensors strength in Provenzano *et al* dataset. **B.** Correlation of splicing index and composite score average of percent predicted quantitative muscle testing of ankle dorsiflexors, knee extensors and grip strength in Provenzano *et al* dataset. **C.** Correlation of splicing index and composite score average of percent predicted quantitative muscle testing of ankle dorsiflexors and grip strength in Provenzano *et al* dataset. **D.** Correlation of splicing index and composite score average of percent predicted quantitative muscle testing of knee extensors and grip strength in Provenzano *et al* dataset. Abbreviations: BL: baseline, SI: splicing index, %: percent
