# Supplementary material for: Three-Year Longitudinal Analysis of Splicing Changes in Myotonic Dystrophy Type 1
Source: bioRxiv. 2026 Oct 1:2026.09.25.754454. Preprint. [Version 1] doi: 10.64898/2026.09.25.754454 (PMC13648444; doi:10.64898/2026.09.25.754454)
Supplement: Supplementary file 2 [file media-2.pdf]

	Delta SI score	% change ankle dorsi	% change knee ext	% change pinch	% change grip	% change composite (ankle dorsi-knee ext)	% change composite (ankle dorsi-knee ext-grip)	% change composite (ankle dorsi-grip)	%change composite (knee ext-grip)
**BL age**	-0.30	0.03	-0.20	-0.27	-0.23	-0.08	-0.11	0.04	-0.20
**BL CTG**	0.17	0.04	-0.12	0.14	-0.12	-0.02	-0.10	-0.13	-0.25
**BL SI score**	-0.18	0.17	-0.34	0.16	-0.42	-0.06	-0.25	-0.12	-0.51*
**BL knee Ext PP**	-0.22	0.12	0.13	-0.01	0.19	0.16	0.25	0.23	0.34
**BL ankle PP**	-0.28	0.24	0.50*	0.45	0.34	0.26	0.39	0.40	0.35
**BL grip PP**	-0.38	0.22	0.52*	-0.04	0.05	0.18	0.19	0.24	0.20
**BL composite (ankle dorsi-knee ext)**	-0.26	0.19	0.34	0.27	0.36	0.26	0.39	0.35	0.43
**BL composite (ankle dorsi-knee ext-grip)**	-0.35	0.36	0.45	0.25	0.21	0.33	0.41	0.43	0.34
**BL composite (ankle dorsi-grip)**	-0.31	0.30	0.54*	0.23	0.16	0.25	0.32	0.36	0.24
**BL composite (knee ext-grip)**	-0.28	0.25	0.42	-0.06	0.18	0.23	0.29	0.30	0.36

-1.0 -0.5 0 0.5 1.0

Supplemental figure 2: Spearman correlations of splicing index and clinical values at baseline and changes over 3 years. *$p<0.05$, abbreviations: BL: baseline, CTG: number of CTG repeats, SI: splicing index, % change ankle dorsi: percent change over 3 years of ankle dorsiflexors strength, % change knee ext: percent change over 3 years of knee extensors strength, % change pinch: percent change over 3 years of pinch strength, % change grip: percent change over 3 years of grip strength, % change composite (ankle dorsi-knee ext): percent change over 3 years of composite score average of percent predicted quantitative muscle testing of ankle dorsiflexors and knee extensors, % change composite (ankle dorsi-knee ext-grip): percent change over 3 years of composite score average of percent predicted quantitative muscle testing of ankle dorsiflexors, knee extensors and grip, % change composite (ankle dorsi-grip): percent change over 3 years of composite score average of percent predicted quantitative muscle testing of ankle dorsiflexors and grip, % change composite (knee ext-grip): percent change over 3 years of composite score average of percent predicted quantitative muscle testing of knee extensors and grip, knee ext PP: knee extensors percent predicted quantitative muscle testing strength, ankle PP: ankle dorsiflexors percent predicted quantitative muscle testing strength, grip pp: grip percent predicted quantitative muscle testing strength, composite (ankle dorsi-knee ext): composite score average of percent predicted quantitative muscle testing of ankle dorsiflexors and knee extensors strength, composite (ankle dorsi-knee ext-grip): composite score average of percent predicted quantitative muscle testing of ankle dorsiflexors, knee extensors and grip strength, composite (ankle dorsi-grip): composite score average of percent predicted quantitative muscle testing of ankle dorsiflexors and grip strength, composite (knee ext-grip): composite score average of percent predicted quantitative muscle testing of knee extensors and grip strength.
