# Supplementary material for: Three-Year Longitudinal Analysis of Splicing Changes in Myotonic Dystrophy Type 1
Source: bioRxiv. 2026 Oct 1:2026.09.25.754454. Preprint. [Version 1] doi: 10.64898/2026.09.25.754454 (PMC13648444; doi:10.64898/2026.09.25.754454)
Supplement: Supplementary file 3 [file media-3.pdf]

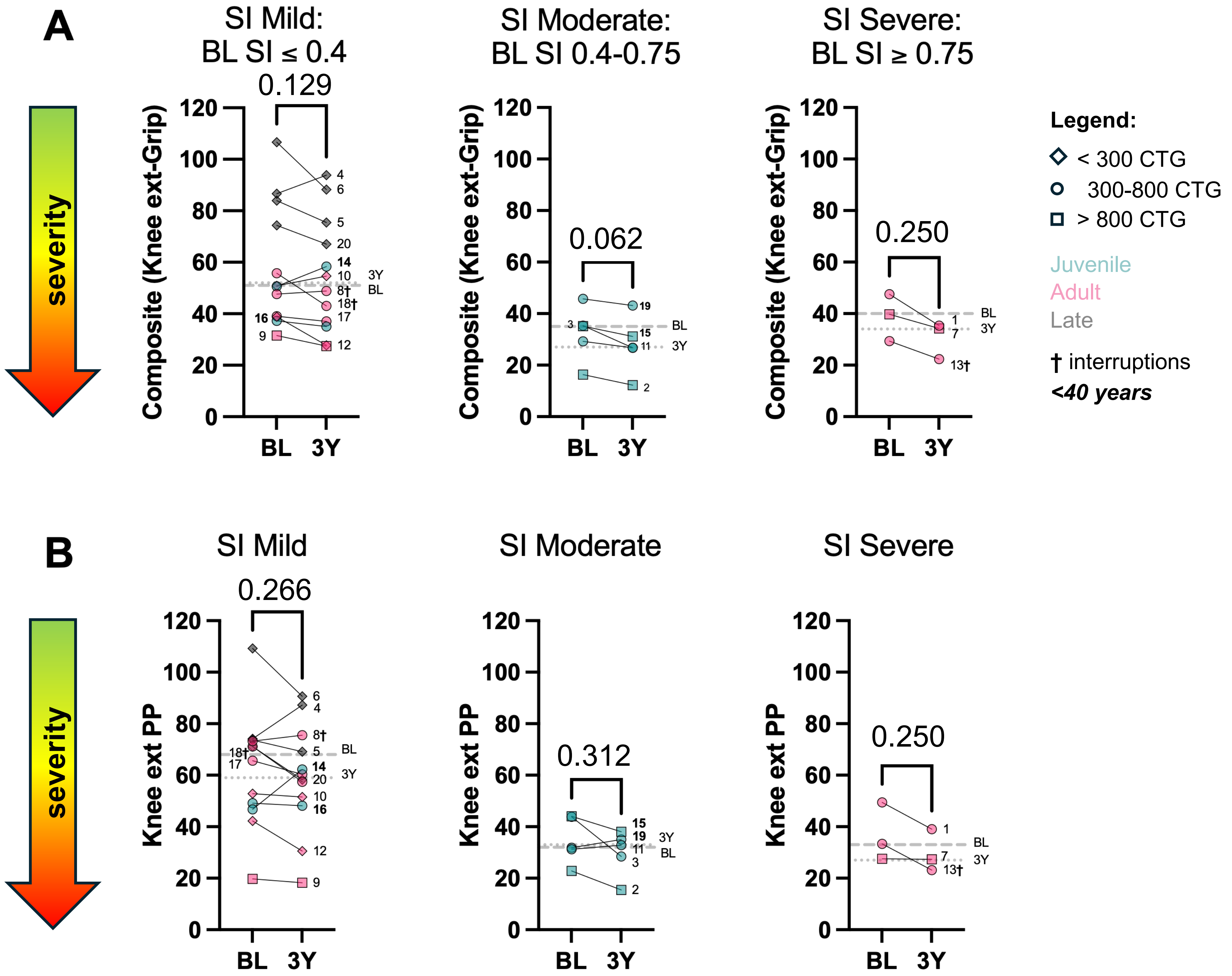

Supplemental Figure 3: composite score of knee extensors and grip strength and knee extensors strength changes over 3 years. A. Composite score of knee extensors and grip strength changes over 3 years. Shapes represent their CTG repeats <300 (diamond), 300-800 (circle), >800 (square) while colors represent their phenotype juvenile (green), adult (pink), late (grey), participants with interruption of their repeats have cross next to their ID in their labels and ***bold in italic*** ID labels are participants <40 years old. Median are presented as grey lines large dash= baseline, smaller dash=3 years. . **B.** Changes in knee extensors strength over 3 years. Shapes represent their CTG repeats <300 (diamond), 300-800 (circle), >800 (square) while colors represent their phenotype juvenile (green), adult (pink), late (grey), participants with interruption of their repeats have cross next to their ID in their labels and ***bold in italic*** ID labels are participants <40 years old. Median are presented as grey lines large dash= baseline, smaller dash=3 years. Abbreviations: SI: splice index, BL: baseline, 3Y: 3 years, composite (knee ext-grip): composite score average of percent predicted quantitative muscle testing of knee extensors and grip strength, knee ext pp: knee extensors percent predicted quantitative muscle testing strength, CTG: number of CTG repeats. *p<0.05 Wilcoxon test.
