# Supplementary material for: Three-Year Longitudinal Analysis of Splicing Changes in Myotonic Dystrophy Type 1
Source: bioRxiv. 2026 Oct 1:2026.09.25.754454. Preprint. [Version 1] doi: 10.64898/2026.09.25.754454 (PMC13648444; doi:10.64898/2026.09.25.754454)
Supplement: Supplementary file 4 [file media-4.pdf]

Supplemental Table 1: Additional regression models of changes in SI score and muscle strength over 3 years

Model	Parameter	Standardized β	p-value	Adjusted R^2	p-value
**Changes in SI score**					
A. Delta SI score ~ BL SI score + BL ankle dorsiflexors strength (% predicted)	BL SI score	-0.510	0.047		
	BL ankle dorsiflexors strength (% predicted)	-0.585	0.026	0.226	0.050
B. Delta SI score ~ BL SI score + BL grip strength (% predicted)	BL SI score	-0.482	0.053		
	BL grip strength (% predicted)	-0.530	0.036	0.191	0.064
C. Delta SI score ~ BL SI score + BL composite score of ankle dorsiflexors-knee extensors strength (% predicted)	BL SI score	-0.683	0.012		
	BL composite score of ankle dorsiflexors-knee extensors strength (% predicted)	-0.757	0.006	0.341	0.014
D. Delta SI score ~ BL SI score + BL composite score of ankle dorsiflexors-knee extensors-grip strength (% predicted)	BL SI score	-0.628	0.016		
	BL composite score of ankle dorsiflexors-knee extensors-grip strength (% predicted)	-0.718	0.007	0.330	0.016
E. Delta SI score ~ BL SI score + BL composite score of ankle dorsiflexors-grip strength (% predicted)	BL SI score	-0.501	0.047		
	BL composite score of ankle dorsiflexors-grip strength (% predicted)	-0.591	0.022	0.239	0.044
**Changes in muscle strength**					
F. % change knee extensors strength ~ BL SI score + BL age	BL SI score	-0.320	0.136		
	BL age	-0.476	0.032	0.216	0.049
G. % change grip strength ~ BL SI score + BL age	BL SI score	-0.440	0.046		
	BL age	-0.365	0.092	0.212	0.051

Abbreviations: delta SI score: difference in SI score over 3 years, BL: baseline, SI splicing index, % : percent
