# Supplementary material for: Three-Year Longitudinal Analysis of Splicing Changes in Myotonic Dystrophy Type 1
Source: bioRxiv. 2026 Oct 1:2026.09.25.754454. Preprint. [Version 1] doi: 10.64898/2026.09.25.754454 (PMC13648444; doi:10.64898/2026.09.25.754454)
Supplement: Supplementary file 5 [file media-5.pdf]

Supplemental Table 2: SI scores

ID	TA normalized BL SI	TA normalized 3Y SI	Changes in TA normalized SI
1	0.980	0.909	-0.072
7	0.895	0.884	-0.01
13	0.770	0.863	0.093
2	0.667	0.749	0.082
11	0.615	0.538	-0.077
15	0.517	0.649	0.132
3	0.464	0.951	0.487
19	0.41	0.464	0.054
8	0.322	0.386	0.064
18	0.238	0.391	0.153
9	0.234	0.714	0.480
12	0.228	1.056	0.827
14	0.206	0.402	0.196
16	0.162	0.416	0.254
5	0.157	0.087	-0.07
17	0.073	0.249	0.177
10	0.042	0.33	0.288
20	-0.024	-0.008	0.016
4	-0.028	-0.016	0.013
6	-0.039	0.011	0.05
Median	0.236	0.440	0.088
Q1, Q3	0.094, 0.591	0.269, 0.835	0.013, 0.240

Abbreviations: TA: *tibialis anterior*, BL: baseline, SI: splicing index, 3Y: 3 years
